# Pleural cryobiopsy is useful for comprehensive cancer genetic panel testing

**DOI:** 10.1002/rcr2.581

**Published:** 2020-05-13

**Authors:** Satoru Ishii, Hiromu Watanabe, Shinyu Izumi, Masayuki Hojo, Haruhito Sugiyama

**Affiliations:** ^1^ Department of Respiratory Medicine National Center for Global Health and Medicine Tokyo Japan

**Keywords:** Cryobiopsy, comprehensive cancer genetic panel testing, pleuroscopy, pleural disease

## Abstract

An 83‐year‐old woman presented with dyspnoea. Her chest X‐ray showed a right‐sided pleural effusion. Flex‐rigid pleuroscopy was performed and showed a mass in the anterior portion. The mass was biopsied with conventional biopsy forceps, but the mass was solid, and sufficient tissues could not be obtained. Therefore, the mass was biopsied with a cryoprobe. The tip of the probe was attached to the mass, and it was cooled with carbon dioxide once for 5 sec and then for 7 sec in the same place. The tissue size obtained was 2 mm by conventional biopsy forceps, and 5 mm at 5 sec and 12 mm at 7 sec by cryobiopsy. Histological analysis of the conventional biopsy forceps specimen showed cancer cells in the glandular cavity, but it was not sufficient tissue for comprehensive cancer genetic panel (CGP) testing. The cryobiopsy specimens showed cancer cells and sufficient tissue for comprehensive CGP testing.

## Graphical abstract

Here, we describe a case of an 83‐year‐old woman in whom insufficient pleural tissue to perform comprehensive cancer genetic panel testing was obtained with biopsy forceps, but sufficient tissue was obtained with cryobiopsy.

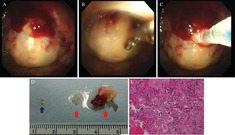

## Introduction

Tissue sampling could originally only establish the diagnosis of lung cancer, but now sufficient tissue sampling is needed for next‐generation sequencing for epidermal growth factor receptor (EGFR), anaplastic lymphoma kinase (ALK), etc. From one tissue sample, comprehensive cancer genetic panel (CGP) testing involving multiple genetic tests at the same time is done in Japan. The samples obtained by conventional biopsy forceps are too small, and have insufficient tissue depth. A cryoprobe is useful to obtain sufficient tissues by bronchoscopy, as well as by flex‐rigid pleuroscopy. In the present case, insufficient pleural tissue was obtained to perform CGP testing with biopsy forceps, but sufficient tissue was obtained with cryobiopsy.

## Case Report

**Figure 1:**
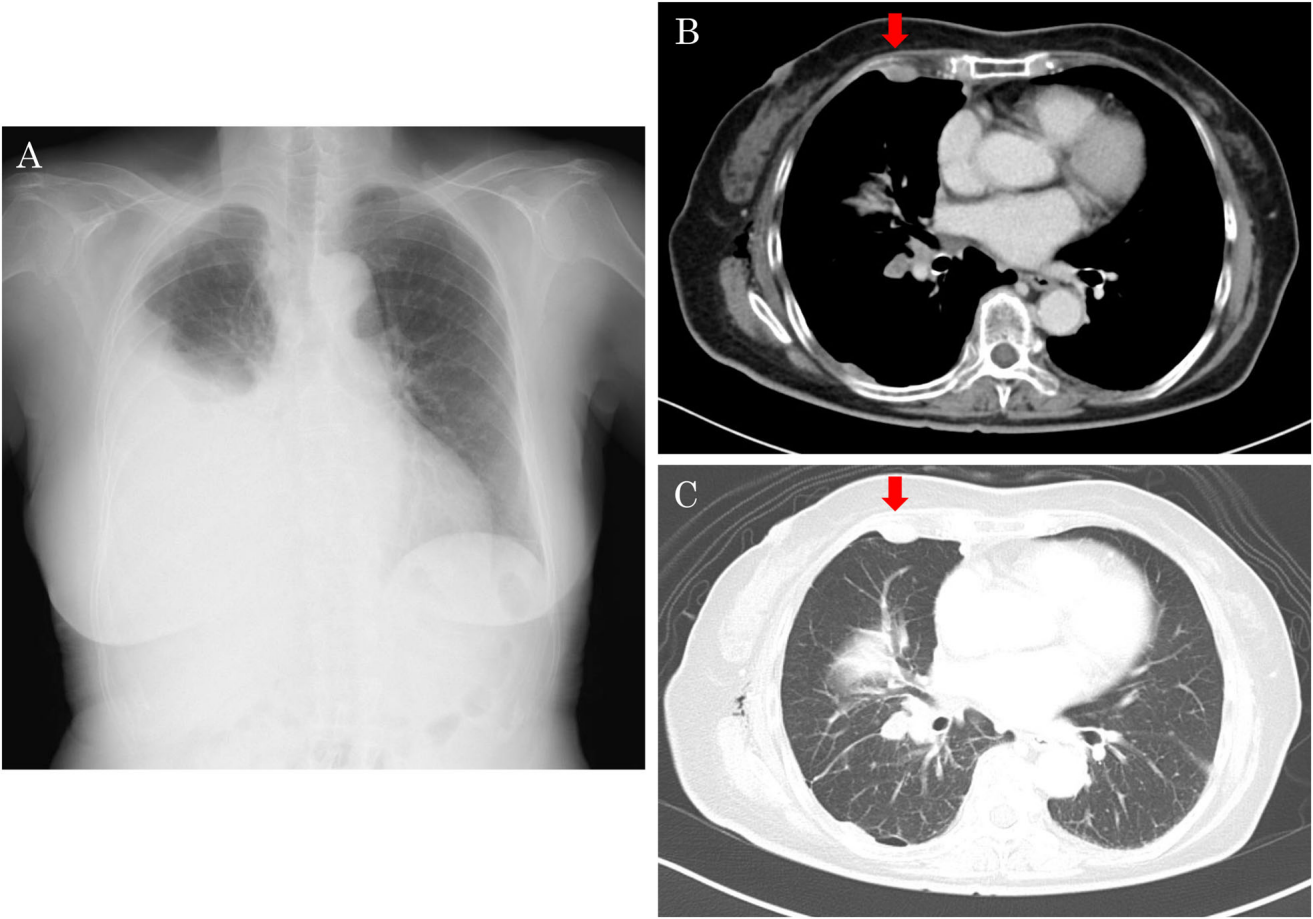
Chest X‐ray shows right‐sided pleural effusion (A). A nodular shadow is also visualized in the anterior portion of chest computed tomography (CT) scans (B, C).

An 83‐year‐old woman presented with dyspnoea. Her chest X‐ray showed a right‐sided pleural effusion (Fig. 1A). A thoracic drain was inserted, and the pleural effusion was drained because she had a peripheral capillary oxygen saturation (SpO_2_) of 93%. A nodular shadow was also visualized in the anterior chest on computed tomography (CT) scans (Fig. 1B, C). Pleural cytodiagnosis was positive for adenocarcinoma. However, the pleural cell block was insufficient for CGP testing. Hence, pleuroscopy under local anaesthesia was performed to gather sufficient tissue for CGP testing.

**Figure 2:**
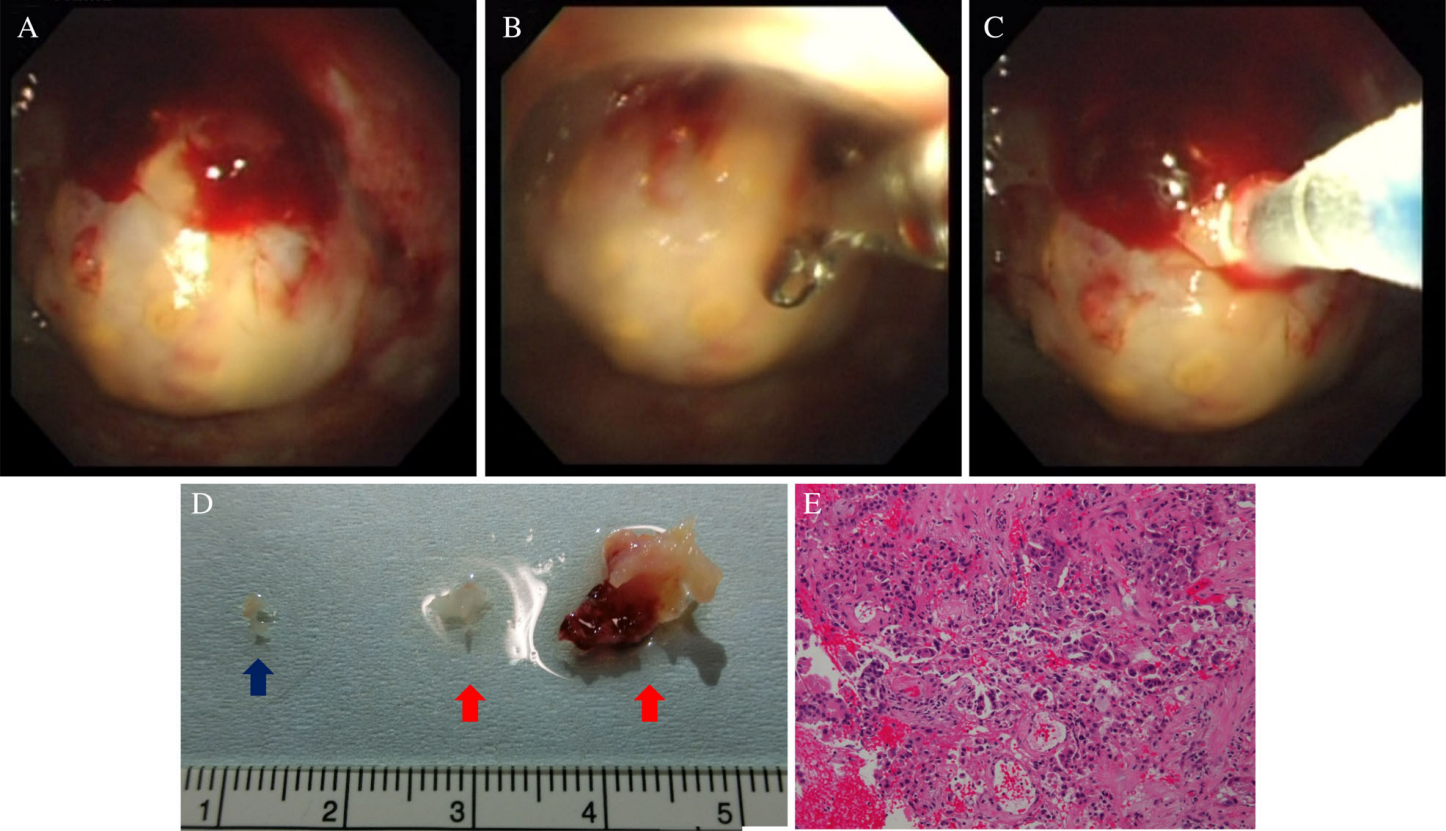
Pleuroscope observation of the mass in the anterior chest (A). Conventional biopsy forceps biopsied the mass, but the mass is solid and the amount of tissue is not sufficient (B). The tip of the cryoprobe was attached to the mass and cooled with carbon dioxide (C). Tissue obtained by conventional biopsy forceps (blue arrow), and tissue at 5 sec (red left arrow) and at 7 sec (red right arrow) obtained by cryobiopsy (D). A histological analysis of the cryobiopsy shows cancer cells in the glandular cavity (E).

Flex‐rigid pleuroscopy was performed using an LTF‐240 (Olympus, Japan). Pleuroscopy showed the mass in the anterior chest (Fig. 2A). The mass was biopsied by conventional biopsy forceps (FB‐231D; Olympus), but the mass was solid, and the tissue samples obtained were insufficient (Fig. 2B). Therefore, the mass was biopsied with a cryoprobe (2.0 mm probe; Erbe Elektromedizin, GmbH, Germany). The tip of the probe was attached to the mass, and it was cooled once with carbon dioxide for 5 sec and then for 7 sec in the same place (Fig. 2C). The frozen tissue sample was extracted by pulling and released from the probe by thawing with normal saline. Slight bleeding was seen in one biopsied part, but it was stanched spontaneously. The tissue size obtained was 2 mm by conventional biopsy forceps, and 5 mm at 5 sec and 12 mm at 7 sec by cryobiopsy (Fig. 2D). Histological analysis of the conventional biopsy forceps specimen showed cancer cells in the glandular cavity, but it was insufficient for CGP testing. The cryobiopsy specimens showed cancer cells, and there was sufficient tissue for CGP testing (Fig. 2E). On CGP, EGFR exon 19 deletion was positive, and the patient was started on osimertinib.

## Discussion

Pleural cytological diagnosis and blind pleural biopsy with thoracentesis are simple procedures for evaluating pleural disease, but the diagnostic yields are reportedly low. Cytological diagnosis is common for carcinomatous pleurisy, but the positive cytology ratio is reported to be only 62%, while that of blind pleural biopsy is 42% and that of pleuroscopy under local anaesthesia is 79–96% [1]. Pleuroscopy with local anaesthesia permits parietal pleural biopsies to be taken under direct vision. It can be performed without the need for intubation or single‐lung ventilation. It is a minimally invasive procedure with a low complication rate and low cost. It has also been reported as the most accurate tool for establishing the diagnosis of carcinomatous pleurisy. Next‐generation sequencing for EGFR, ALK, and so on is required in current cancer diagnosis. It has been possible to perform CGP testing since 2019 in Japan. These CGP assays generally require a minimum tumour cell content of 20–30% to have an adequate sensitivity for all classes of alterations [2, 3]. A larger tumour cell size improves the overall impression of the tumour cell content. Pleural cryobiopsy is safe and consistently yields larger tissue specimens than flexible forceps biopsy. Chen et al. reported cryobiopsy tissue samples that were significantly larger (9.4 ± 4.9 mm) than those obtained by forceps biopsy (4.2 ± 2.3 mm) [4]. Immunohistochemical staining revealed 98.9% feasible tissue in cryobiopsy samples, more than 87.0% in forceps biopsy samples. Cryobiopsy results in fewer instances of crush artefact than forceps biopsy [5, 6, 7]. Rozman et al. reported cryobiopsy tissues were of good quality, with the level of artefacts below 25%. The tissues were adequate for histological diagnosis, immunohistochemical staining, and DNA isolation [5]. Sharfiq et al. reported their review of 311 cryobiopsy procedures, and major bleeding was not reported in even one case [6]. Dhooria et al. reported that the diagnostic yield was not different between cryobiopsy and forceps biopsy [7]. In the present case, the surface of the tumour was solid, and biopsy forceps could not obtain sufficient tissue, but the cryoprobe was useful for obtaining sufficient tissue for CGP testing. There is a possibility that the detection rate of CGP testing differs between cryobiopsy and forceps biopsy, and this needs to be taken into consideration.

### Disclosure Statement

Appropriate written informed consent was obtained for publication of this case report and accompanying images.
